# The Effects of Laparoscopic Sleeve Gastrectomy on Body Mass Index (BMI) and Glycated Hemoglobin (HbA1c) Levels

**DOI:** 10.7759/cureus.70695

**Published:** 2024-10-02

**Authors:** Khalid M Alayed, Ahmed M AlKhawashki, Abdulmalik M Mokhtar, Raghad A Alnafisah, Khawla A Alammari, Malak F Alsharif

**Affiliations:** 1 Internal Medicine, King Saud University Medical City, Riyadh, SAU; 2 College of Medicine, King Saud University Medical City, Riyadh, SAU

**Keywords:** bariatric surgeries, bariatric surgery and diabetes, body mass index (bmi), diabetes treatment, endocrinology and diabetes, glycated hemoglobin (hba1c), kingdom of saudi arabia (ksa), laparoscopic sleeve gastrectomy (lsg), type 2 diabetes, weight reduction

## Abstract

Background and significance

Bariatric surgery is an effective surgical intervention for weight loss and metabolic improvement. Articles tackling obesity and bariatric surgery with its preoperative preferences and postoperative findings are needed. From that stance, we aim to accurately document the impact of bariatric surgery, particularly laparoscopic sleeve gastrectomy (LSG), on body mass index (BMI) and glycated hemoglobin (HbA1c) levels.

Patients and methods

We present a retrospective cohort study conducted on 111 LSG patients from a total of 1633 patients who underwent bariatric surgery from January 23, 2018, to December 31, 2019, at King Saud University Medical City in Riyadh, Saudi Arabia. Patients were divided into three groups: nondiabetics, prediabetics, and diabetics. For each group, demographic characteristics as well as preoperative and postoperative BMI and HbA1c values were collected.

Results

The mean patient age was 41.35±11.8 years, with 56.8% being female. Our analysis showed that BMI values for all three groups had a significant and nearly similar overall decrease in value postoperatively (mean difference: 14.43, p<0.001). HbA1c levels also significantly improved, with the largest reduction seen in the diabetic group (from 8.7±1.5 to 6.6±1.4, p<0.001), followed by the prediabetic group (from 5.9±0.2 to 5.4±0.3, p<0.001) and the nondiabetic group (from 5.4±0.1 to 5.2±0.3, p=0.003).

Conclusion

LSG leads to significant improvements in BMI and HbA1c levels. Postoperatively, diabetic patients showed the greatest reduction in HbA1c percentage, supporting LSG's role in enhancing metabolic health.

## Introduction

Obesity is a major public health challenge in the Middle East particularly the Kingdom of Saudi Arabia. The alarming rise in the prevalence of obesity is paralleled by an increased burden of chronic noncommunicable diseases, especially type 2 diabetes mellitus (T2DM), hypercholesterolemia, and hypertension [1]. Obesity is in high association with serious health conditions and comorbidities including metabolic syndrome [2], cardiovascular diseases [3], sleep apnea, hyperlipidemia, and both type 1 diabetes mellitus and T2DM [4-6], therefore paving the way for bariatric surgery to solidify its position among other remedies and treatments for obesity [7,8]. 

It has been shown that by undergoing bariatric surgery, obese patients who suffer from diabetes can achieve marked reductions in weight and body mass index (BMI) values as well as notice improvements in their glycemic status, including their postoperative levels of glycated hemoglobin (HbA1c) and fasting plasma glucose (FPG) [9,10]. HbA1c is the measurement of average blood glucose concentrations over a two- to three-month period, which is the average lifespan of a red blood cell. The role that HbA1c plays is extremely crucial especially in monitoring glycemic control and the diagnosis and treatment of diabetes [11,12].

Bariatric surgery has revolutionized the treatment of both obesity and T2DM, as it begets important improvements in areas like insulin sensitivity and the general quality of life of suffering patients [13,14]. Bariatric surgery can also improve T2DM status by mainly halting the development of microvascular complications such as nephropathy and retinopathy. Macrovascular diseases related to T2DM, including coronary artery disease and cerebrovascular disease, can also be stopped or corrected by the performance of bariatric surgery especially in obese patients [15-17]. 

The surgical assumptions around the remission of T2DM should be adjusted and tailored more towards glycemic control and weight reduction outcomes. Remission of T2DM depends on the type of surgery performed, with greater weight loss correlating with better resolution of symptoms [18,19]. The value of bariatric surgery should always depend on whether the potential benefits make the risks such as poor postoperative nutrition acceptable [20,21]. Like in any other life-changing case, before considering bariatric surgery, patients are given the option of adherence to lifestyle changes with medical management. For obese patients with T2DM, combining both treatment options yields a higher possibility of reaching the desired goal or target. When lifestyle changes or medical management don't give the desired outcomes or have very minimal results, then mainly bariatric surgery alone can result in the wanted improvements in weight loss, diabetes control, or the remission of T2DM [20,22]. Both bariatric surgery and intensive lifestyle changes are effective in controlling or stopping T2DM, but the majority of patients choose the surgical option due to their difficulties in properly managing and sustaining both their blood glucose levels and blood pressure values [23,24]. Lipid profile-wise, both treatment options achieve desired outcomes, but when put side to side, the surgical option yielded a significant increase in the level of high-density lipoprotein (HDL) and a significant decrease in both triglycerides and low-density lipoprotein particles (LDL-P) [25-27].

Due to the aforementioned reasons, it is extremely crucial to highlight and clearly define the trends of bariatric surgery in the region as well as accurately document the noticed preoperative and postoperative patterns.

## Materials and methods

This retrospective cohort study was conducted on bariatric surgery patients at King Saud University Medical City in Riyadh, Saudi Arabia. The study was approved by the Institutional Review Board of King Saud University on October 18, 2022 (approval number: E-22-7192; reference number: 22/0776/IRB). 

**Figure 1:**
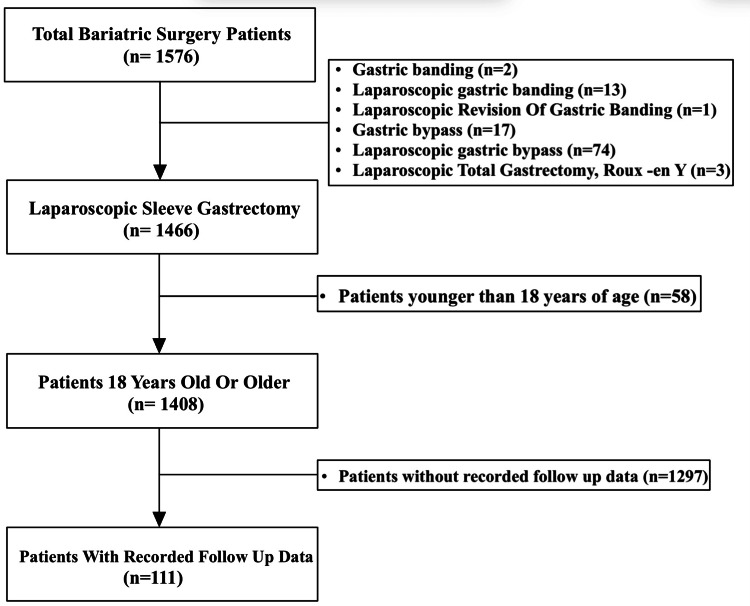
Flowchart showing patients included in the present study

Furthermore, data was retrieved from the medical records of patients who underwent bariatric surgery, no identifiers were used, and privacy was ensured. Patients' characteristics and baseline data were collected including age, gender, type of surgery, BMI, HbA1c, and diabetic status. BMI and HbA1c levels were collected both preoperatively and a year postoperatively. Ideally, a one-year follow-up period should be achieved, but not all patients had records of exactly one year, so we calculated a mean follow-up period of 10.5 months for all patients. Patients were included if they had sufficient preoperative and postoperative recorded data and were above 18 years of age. As per Figure 1, from a total of 1633 patients who underwent bariatric surgery between January 23, 2018, and December 31, 2019, only 111 patients satisfied our inclusion criteria. In terms of the type of bariatric surgery, all 111 patients underwent laparoscopic sleeve gastrectomy (LSG). Patients under the age of 18, patients who had no follow-up data, and patients with missing records of BMI and/or HbA1c were excluded. 

In addition to that, we divided the patients into three groups: nondiabetics, prediabetics, and diabetics. As per the American Diabetes Association criteria [28], nondiabetics were those with baseline HbA1c levels less than 5.7%, and prediabetics were those with baseline HbA1c levels from 5.7% to 6.4%, whereas diabetics were those who had a baseline HbA1c greater than or equal to 6.5%. Differences between the preoperative visit and postoperative visit were calculated for each reading of BMI and HbA1c. 

Data analysis

Data analysis was curated using IBM SPSS Statistics for Windows, Version 26.0 (Released 2019; IBM Corp., Armonk, New York, United States). Frequencies and percentages were calculated for categorical variables. For quantitative or continuous data, we presented them as mean±standard deviation. A paired sample t-test was used to calculate and compare the preoperative and postoperative HbA1c and BMI means in the nondiabetic, prediabetic, and diabetic groups. A p-value of ≤0.05 was used to report the statistical significance of the results. 

## Results

Our study included a total of 111 patients who underwent LSG from January 23, 2018, to December 31, 2019, and fulfilled the inclusion criteria. The characteristics of patients are presented in Table 1. The mean age was 41.35±11.8 with an age range from 18 to 68 years, and more female patients (56.8%) were documented. Preoperatively, 16.2% of our patients were nondiabetic, 40.5% were prediabetic, and the majority of patients (43.2%) were diabetic. In terms of the diabetic status postoperatively, the majority of patients became nondiabetic (52.3%), followed by prediabetic (30.6%) and then diabetic (17.1%).

**Table 1:** Characteristics of patients (n=111) n: total number of patients; BMI: body mass index; HbA1c: glycated hemoglobin

Variable	Number of patients (%)
Gender	
Male	48 (43.2%)
Female	63 (56.8%)
Diabetic status preoperatively	
Nondiabetic	18 (16.2%)
Prediabetic	45 (40.5%)
Diabetic	48 (43.2%)
Diabetic status postoperatively	
Nondiabetic	58 (52.3%)
Prediabetic	34 (30.6%)
Diabetic	19 (17.1%)
Variable	Mean±standard deviation
Age (18-68) years	41.35±11.8
Preoperative BMI kg/m^2^	44.52±7.64
Postoperative BMI kg/m^2^	30.09±5.48
Preoperative HbA1c %	7.06±1.78
Postoperative HbA1c %	5.91±1.13

The paired samples t-test comparison between the preoperative and postoperative BMI values and HbA1c % for all 111 patients is detailed in Table 2. A statistically significant difference between the preoperative and postoperative BMI values (mean differences: 14.43, p<0.001) has been found. The comparison between preoperative and postoperative HbA1c % also yielded a statistical significance (mean differences: 1.15, p<0.001).

**Table 2:** Paired samples t-test of patients' preoperative and postoperative BMI values and HbA1c % (n=111) n: total number of patients: SD: standard deviation; BMI: body mass index; HbA1c: glycated hemoglobin; ***: statistically significant

Variable	Preoperative mean±SD	Postoperative mean±SD	Mean differences	P-value
BMI (kg/m^2^)	44.52±7.64	30.09±5.48	14.43	<0.001***
HbA1c %	7.06±1.78	5.91±1.13	1.15	<0.001***

**Figure 2:**
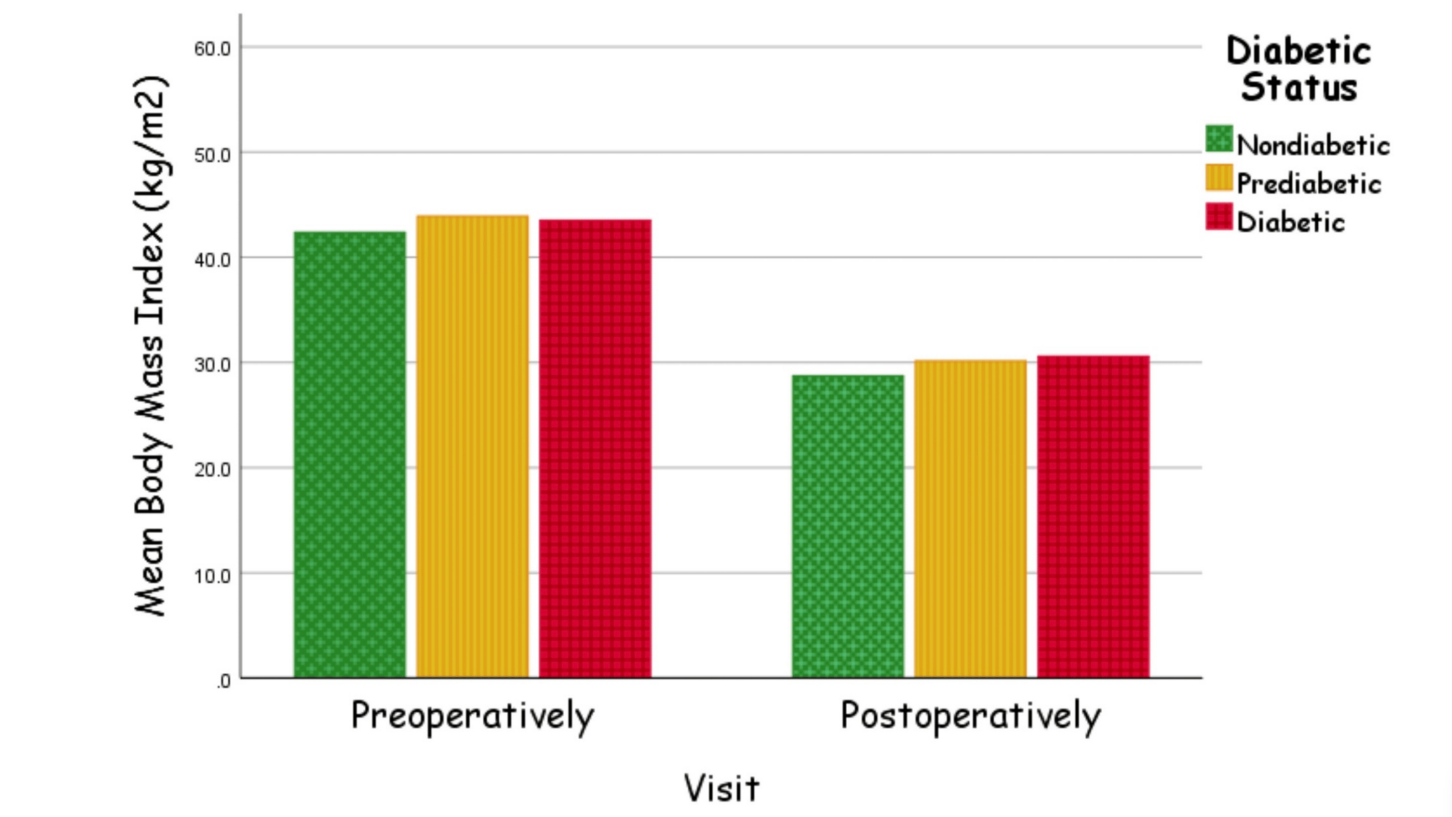
BMI value pre-/postoperatively BMI: body mass index

As displayed in Figure 2, our BMI value analysis was filtered further by the patients' diabetic status in which we noticed significant and nearly similar reduction in all three diabetic status groups. The BMI values for the nondiabetic group were 42.3±7.5 preoperatively versus 28.7±5.5 postoperatively (p<0.001). The BMI values for the prediabetic group were 43.9±8.1 preoperatively versus 30.1±5.4 postoperatively (p<0.001). Lastly, the diabetic group had 43.5±7.1 preoperatively versus 30.5±5.5 postoperatively (p<0.001) for their BMI value reduction.

**Figure 3:**
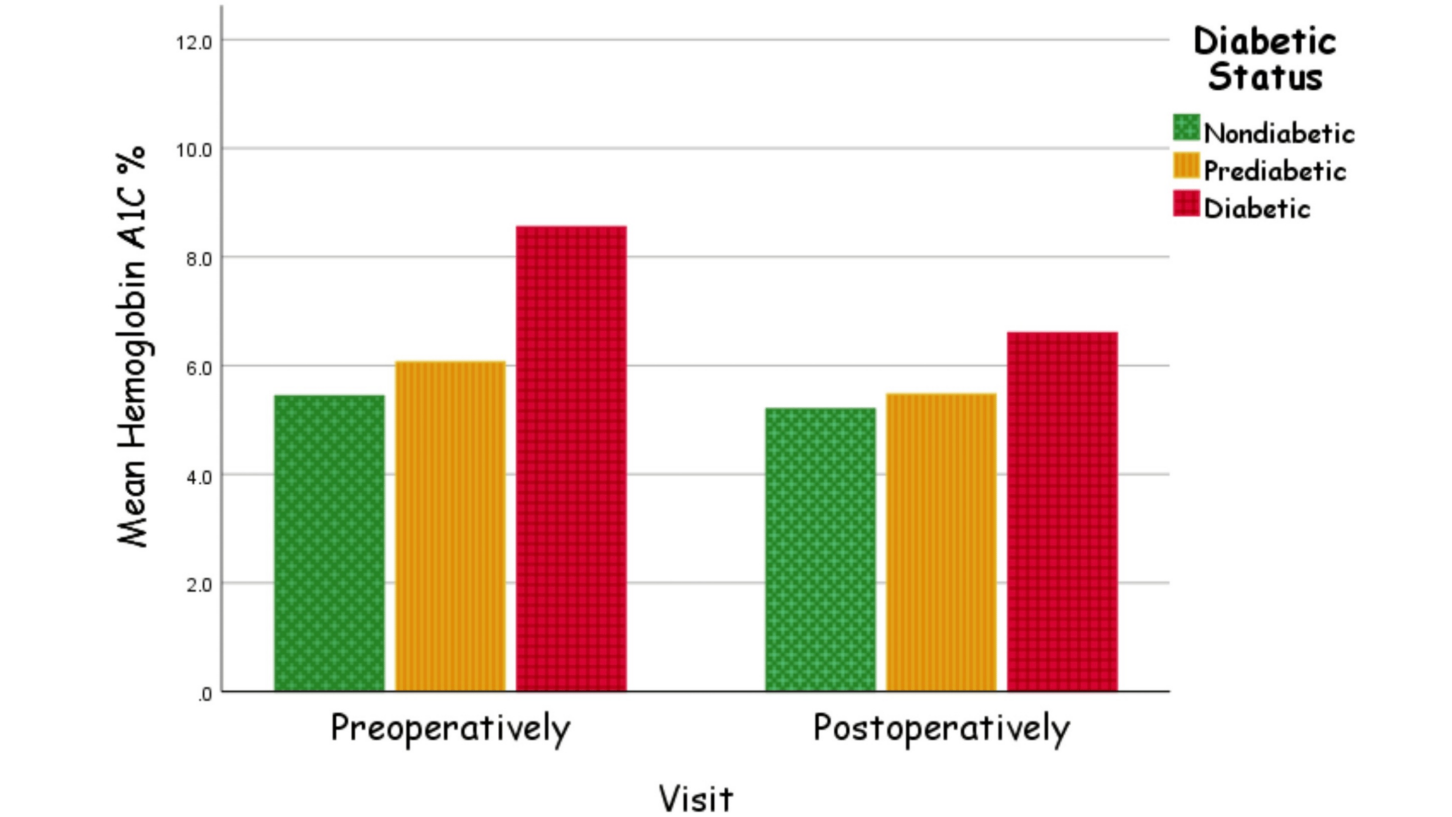
HbA1c % pre-/postoperatively HbA1c: glycated hemoglobin

In Figure 3, our analysis for HbA1c % was also categorized further by the diabetic status, where, in the nondiabetic group, the slightest but still significant reduction in HbA1c % (5.4±0.1 preoperatively versus 5.2±0.3 postoperatively, p=0.003) was noticed. As for the prediabetic group, a moderate but significant reduction in HbA1c % (5.9±0.2 preoperatively versus 5.4±0.3 postoperatively, p<0.001) was documented. Finally, the diabetic group showed the greatest improvement (8.7±1.5 preoperatively versus 6.6±1.4 postoperatively, p<0.001) while having statistical significance.

## Discussion

We retrospectively gathered recorded data on LSG patients at King Saud University Medical City in Riyadh, Saudi Arabia, between January 23, 2018, and December 31, 2019. LSG has been shown to significantly reduce BMI and HbA1c, confirming its efficacy. Contemporary research and the increasing amount of evidence in the literature suggest that bariatric surgery is an effective treatment option for T2DM in obese patients, as its yields are superior to that of medical interventions [9,29,30]. As of now, the largest randomized controlled trial with the longest follow-up time is the Surgical Treatment and Medications to Potentially Eradicate Diabetes Efficiently (STAMPEDE) trial. In the STAMPEDE trial done by Schauer et al., the favorable effects of bariatric surgery on glycemic control were found to be quite reliable and also resulted in a persistent reduction in the utilization of diabetes and cardiovascular medication. They also reported that patients who received solely the medical intervention had a slight reduction in HbA1c levels, compared to the surgery with medical intervention patients where HbA1c was significantly reduced [30].

Previous literature also reported that substantial reduction in BMI occurs post-surgical interventions. Mingrone et al. found that variables like BMI and waist circumference in surgical patients showed better results than in intensive medical intervention patients [29]. Our BMI analysis as displayed in Figure 2 showed that the nondiabetic, prediabetic, and diabetic groups all had significant and nearly similar overall reduction in BMI values after the LSG procedure. The findings of our analysis are consistent with the results of other studies that have investigated the effects of LSG on BMI. As reported in Al-Madinah, Saudi Arabia, Zaki et al. found that in 10 months following LSG, BMI values dropped (30%) from their baseline prior to surgery [9]. All mentioned findings emphasize the importance of considering surgical options as a viable and potentially more effective means of addressing obesity and its associated health concerns.

Furthermore, Zaki et al. found that patients following LSG had a significant reduction in HbA1c levels (26.4%) compared to their preoperative levels [9]. In Brazil, Geloneze et al. observed that prior to undergoing bariatric surgeries including LSG, the higher the patient's levels of fasting glucose and HbA1c % measured, the greater their levels drop postoperatively [6]. In agreement with local and international studies [6,9], we found that in terms of HbA1c changes, all our three groups showed a statistically significant reduction in HbA1c levels postoperatively, but diabetics demonstrated the greatest improvement. These findings further contribute to the growing body of evidence supporting the efficacy of LSG as an intervention for individuals with obesity and T2DM.

Even though LSG is an effective weight loss remedy, it is not without complications. Nutritional deficiencies, staple line bleeding, and leaking are encountered postoperatively [31-33]. Deficiencies in vitamins B12 and D, iron, and zinc are common due to impaired absorption, requiring long-term monitoring and supplementation [31]. For staple line leaks, early detection and appropriate management, including conservative or surgical options, are crucial for minimizing complications [32,33].

In our study, the clear absence of recorded follow-up care data for the copious number of bariatric surgery patients highlights a critical gap in the management of the healthcare infrastructure. From our findings, the majority of patients seem to only undergo the designated bariatric surgery and then proceed to not attend future follow-up appointments afterwards, thus the large number of no records as per Figure 1. Establishing a national healthcare database could be a significant step towards addressing this issue by providing a centralized platform for tracking and managing patient information. A national healthcare database would also connect primary, secondary, and tertiary healthcare centers together, decreasing waiting times and facilitating ease of follow-up access for patients especially for those affected by distance and crowdedness. It would also empower healthcare providers with the data and information they need to make informed decisions and improve all different treatment outcomes.

Strengths and limitations

Selection bias may limit the ability to generalize the data, as it was collected from patients of a single medical city. Also, the study was conducted over a relatively short period. Another limitation is not taking into consideration the patients' use of anti-diabetic medications in our analysis. It is possible that medication usage could have influenced glycemic control outcomes, and future studies should consider this factor when evaluating the impact of bariatric surgery on diabetes management. Despite these limitations, we managed to shed light on the clinical consequences of bariatric surgery particularly LSG on obtaining optimal glycemic control and weight reduction.

## Conclusions

Both BMI values and HbA1c levels were reduced after LSG. A significant reduction in BMI values postoperatively was noted in all three groups. Postoperatively, diabetics showed the greatest reduction in HbA1c % while having statistical significance. With the clear absence of adequate follow-up data, establishing a national healthcare database that connects centers is imperative as it will ease healthcare access and enhance the management of patient information.
